# The Iron Hand of the State

**Published:** 1909-07-31

**Authors:** 


### Nursing Administration.

### THE IRON HAND OF THE STATE.

The present condition of the agitation in favour of the State registration and State examination of nurses was exposed with great distinctness during the meetings held last week. As regards opinion in England, evidence was produced that sixty-seven matrons in London and one hundred and seventy-five matrons in the provinces were definitely opposed to it. Among the seventy-seven major training schools for nurses in London and in the provinces, only about sixteen matrons support the movement in favour of State registration. When the state of affairs is examined in other countries the position of the movement is seen to be even more forlorn.

In no country can direct progress be pointed to as the result of State registration. In America, where in some of the States success has attended an agitation conducted on somewhat similar lines to that with which English nurses are familiar, registration appears to be responsible for a distinct falling off in the length of training and in the desire of women to embrace the career of nursing. It is singular that a lady from New York, where the term of training prescribed by law is only two years, should venture, in addressing English nurses, to allude to English matrons as "opposed to meet the needs of suffering humanity."

Suffering humanity requiring the ministrations of well-trained nurses might do worse than seek them in the training schools of these same matrons, free from the paralysing action of the "iron hand": training schools where a probationer, instead of being rushed through a complicated and showy curriculum in two years under the influence of hasty legislation, is not considered capable of doing nursing duties until her character has been moulded and her powers fully developed by a course of training extending always to three and often to four years. So far from proving in the States an instrument of progress, State registration is practically proving a death-blow to the three-year system of training which was being welcomed by superintendents and managers previous to legislation. For wherever a State curriculum is established, the institutions preparing candidates for its examinations are forced down to a level low enough for the majority. The congress found an eloquent supporter of registration in Fräulein Karll. But what are the facts? In Germany the prescribed term of training is one year. The delegates from Sweden and Holland, invited to this country in order to bless registration, declared themselves emphatically opposed to it. In both countries they declared that nursing was not yet "ripe" for legislation, and by that very declaration they proclaimed the fact that registration does not in effect encourage progress, but invariably puts impediments in its way. It is least harmful where progress is not desired, and where the only aim is to crystallise a condition of affairs deemed practically perfect, and it is for this reason that heads of government departments and municipal institutions where rigid uniformity is the first desideratum are disposed to favour it.

Throughout the conference on the "International Standard of Nursing," State registration, State examinations, and the uniformity inevitably following in their train were constantly appealed to as a counsel of perfection and a symbol of progress, but nothing is plainer in the whole debate than the fact that State regulations have never done anything to improve the status or the training of nurses. In certain British colonies in which hospitals are State supported and State controlled a system of examination and registration has been carried out by those to whom the governing bodies of these institutions are responsible. The State, governing and supporting the hospitals, controls the conditions under which nurses are trained in them. The Local Government Board might in like manner very easily establish a pass examination in the Poor Law institutions of this country, and refuse to promote any nurses who failed in getting through. Who does not understand that this is a very different thing from invoking a heterogeneous Board to prescribe a hard-and-fast curriculum for nurses trained under every possible kind of condition, in institutions presenting every variety of administration, and governed by bodies chosen from among those who themselves find the money for their support?

The meeting with curious blindness of perception, failed to take into account one of the main factors in the situation, which is that the training of nurses cannot be regarded *per se*. Those who train nurses, still less the nurses who are trained, are not in a position to make decrees. If nurses desire to hand themselves over to the "iron hand of the State," would it not be prudent first to discover whether the supporters and representatives of the enormously costly institutions on which their training depends are like-minded? The training of nurses cannot be carried out in a college: it presupposes the upkeep of institutions of world-wide importance. Nurses are, after all, but one strand in a very complex system for the relief of suffering humanity, and it is idle for certain members of the profession to meet and pass resolutions as though their interests alone were to be regarded in a measure which would set up an outside body established by Parliament, capable of exercising a totally incalculable effect on voluntary institutions.

Without the absolute freedom characteristic of the voluntary charities of this country, it is hardly conceivable that the mighty achievements in medicine and surgery, no less than in nursing, during the last fifty years could have been attained. Is it reasonable to suppose that with the garnered sheaves in full view, with a sense of accomplishment cheering them on to fresh victories, the supporters of these charities, and those medical and nursing superintendents who have lent their lives to the struggle, should voluntarily fetter their action and hand over the control of the great pioneer nursing schools to a Board compelled to study the limitations of inferior establishments?

